# Knowledge-Guided Framework for Synthesizing Contrast-Dependent Data from Multi-Sequence Non-Contrast MRI

**DOI:** 10.3390/diagnostics16040576

**Published:** 2026-02-14

**Authors:** Jinwei Dong, Yihua Chen, Nuoxi Li, Yaqiong Zheng, Guibin Lin, Xingtao Lin, Wangbin Ding

**Affiliations:** 1 College of Physics and Information Engineering, Fuzhou University, Fuzhou 350116, China; 211110003@fzu.edu.cn (J.D.); 241127097@fzu.edu.cn (G.L.); 231110040@fzu.edu.cn (X.L.); 2 School of Medical Imaging, Fujian Medical University, Fuzhou 350122, China; 18859562982@fjmu.edu.cn (Y.C.); 18805902252@163.com (N.L.); zhengyaqiongfjmu@163.com (Y.Z.)

**Keywords:** MRI synthesis, lesion-aware generation, perfusion mapping, multimodal fusion

## Abstract

**Background**: Contrast-enhanced magnetic resonance imaging (MRI), including late gadolinium enhancement (LGE) and cerebral blood volume (CBV) maps, is essential for characterizing pathologies such as myocardial scars and brain tumors. However, acquiring these images requires gadolinium-based contrast agents (GBCAs), which are contraindicated in certain patient populations. Although deep learning enables cross-modality image translation, current methods frequently fail to preserve lesion details, limiting their clinical utility. **Methods**: We propose KGSynth, a knowledge-guided framework designed to synthesize contrast-enhanced MRI from non-contrast sequences. This approach incorporates a knowledge estimator to extract lesion and anatomical features, paired with a style mapping network to capture contrast-specific visual characteristics. By explicitly modeling these distinct components, the framework aims to improve pathological fidelity in the synthesized images. **Results**: Extensive validation on cardiac and brain MRI datasets indicates that KGSynth outperforms existing competing methods. In cardiac LGE synthesis, the model achieved an SSIM of 0.567 and PSNR of 19.48 dB. Similarly, for quantitative brain CBV map synthesis, it yielded an SSIM of 0.697 and PSNR of 24.49 dB. Notably, the method demonstrated improved accuracy in delineating myocardial infarctions and tumor regions compared to baseline models. **Conclusions**: Integrating explicit knowledge guidance into generative models effectively produces diagnostic-quality images without GBCAs. KGSynth preserves pathological accuracy, offering a viable solution for virtual contrast enhancement. This approach holds promise for clinical workflows, particularly for patients with contraindications to contrast agents.

## 1. Introduction

Magnetic resonance imaging (MRI) is a crucial tool in modern medical diagnosis, providing clear visualization of soft tissues without the use of ionizing radiation [1]. The use of gadolinium-based contrast agents (GBCAs) further enhances its diagnostic capabilities, allowing physicians to visualize physiological and pathological changes that would otherwise be difficult to detect [2,3,4]. For example, in cardiology, delayed gadolinium enhancement (LGE) imaging has become the gold standard for assessing myocardial viability and examining scar tissue, providing critical information for patient assessment and treatment planning [5,6]. Similarly, in neuro-oncology, cerebral blood volume (CBV) maps obtained through dynamic contrast-enhanced (DCE) scans are essential for determining tumor grade, differentiating disease progression, and monitoring treatment effectiveness [7,8].

Despite the significant clinical value of GBCAs, their use is subject to certain limitations [9,10,11]. For patients with severe kidney disease, the use of these contrast agents carries the risk of renal systemic fibrosis and is therefore contraindicated. Furthermore, due to concerns about gadolinium deposition in the brain and other tissues, their use requires extreme caution in patients requiring frequent examinations, such as those with chronic diseases or undergoing cancer monitoring [12]. These clinical challenges require researchers to find alternative methods to obtain the same enhanced magnetic resonance imaging diagnostic information without the use of contrast agents [13,14].

To address this issue, deep learning has brought about a new paradigm shift. It can directly use non-contrast MRI scans to synthesize realistic contrast-enhanced images, a process known as virtual contrast enhancement [15,16]. This field is based on advances in generative image transformation approaches, among which approaches like generative adversarial networks (GANs), such as pix2pix [17] and CycleGAN [18], have achieved success in transforming between different image modalities [19,20]. Researchers have also developed specialized virtual contrast generation methods, such as VNE-Net [21], for synthesizing cardiac LGE images from balanced steady-state free precession cine (C0) and T2-weighted (T2w) sequences and various deep learning architectures for generating synthetic gadolinium-enhanced T1-weighted (T1w) brain images to aid in tumor diagnosis [22,23,24]. Our work expands this promising research direction, processing not only static contrast-enhanced images but also quantitative functional maps extracted from dynamic perfusion data.

However, despite these promising developments, many existing synthetic models still struggle to guarantee the accuracy of lesion generation. These models typically maintain the integrity and consistency of the overall anatomical structure but may fail to accurately generate subtle yet crucial features of the lesion tissue. This tendency to ignore lesion characteristics can lead to underestimating the extent of myocardial scarring or misrepresenting complex enhancement patterns of brain tumors, thus reducing the diagnostic reliability of synthetic images [25]. Recognizing this deficiency, more complex methods have emerged. For example, methods like PSCGAN [26] employ progressive strategies to improve myocardial scar generation, often requiring prior knowledge such as segmentation masks. While offering improvements, these methods themselves suffer from complexity, high computational cost, and training difficulties. More importantly, the vast majority of models still use only a single input sequence, creating an information bottleneck and ignoring the rich supplementary information in other conventional sequences.

To address the aforementioned shortcomings, we propose a knowledge-guided synthesis framework, KGSynth. This is a novel approach designed to generate high-quality, high-fidelity contrast-enhanced images, with a particular focus on the accuracy of pathological details. The main hypothesis is that modeling anatomical and lesion knowledge separately significantly improves pathological accuracy in contrast synthesis. KGSynth achieves high-fidelity synthesis of pathological details by establishing a holistic framework that leverages multiple complementary non-contrast MRI sequences to extract prior anatomical and pathological knowledge. Its design incorporates three core innovations: (1) It possesses a dedicated knowledge estimator that automatically identifies and locates potential lesion and shape information from non-contrast sequences, providing knowledge guidance for the synthesis process. (2) It has a synthesis generator that intelligently fuses lesion information, structural information, and contrast patterns to generate reliable contrast-enhanced sequences. (3) We comprehensively tested KGSynth on two distinct and challenging clinical tasks: cardiac LGE synthesis and brain CBV map synthesis. The results demonstrate that KGSynth significantly outperforms existing methods, demonstrating superior accuracy and detail generation in pathology.

## 2. Method

**Figure 1:**
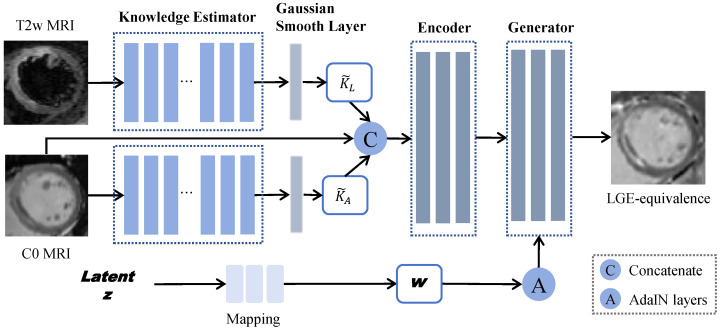
The architecture overview of KGSynth for synthesizing virtual LGE images. A knowledge estimator extracts anatomy and lesion knowledge from the C0 and T2w sequences, respectively. This prior knowledge and C0 are fused and fed to an encoder. Simultaneously, a style mapping network generates style codes from a latent vector. The generator then integrates the knowledge features from the encoder with the style codes via AdaIN layers to produce the final synthesized LGE image. A similar pipeline is employed for CBV map synthesis, where T2w and T1 MRI sequences are leveraged as inputs to generate synthetic CBV maps.

The architecture of KGSynth, as illustrated in Figure 1, comprises three main modules: a knowledge estimator, a conditional encoder generator network, and a style mapping network. The knowledge estimator functions as the core content-aware module, distilling essential priors from multi-sequence non-contrast input images. These priors are then explicitly smoothed and encoded to serve as the conditional guidance for an alias-free generator. Concurrently, the style mapping network transforms a latent vector into style codes that modulate the synthesis process. This design ensures that the synthetic images are both content-faithful and stylistically diverse, while maintaining equivariance to translation and rotation.

### 2.1. Knowledge Estimator

The knowledge estimator is designed to distill relevant anatomical and lesion-related information from non-contrast multi-sequence inputs. Taking cardiac image synthesis as an exemplary application, KGSynth utilizes C0 and T2w sequences (i.e., $I_{C0}$ and $I_{T2w}$) to synthesize the target LGE image.

Each input sequence is processed by a dedicated prior extractor:

- Anatomy knowledge estimator ($\Phi_{A}$): We employ a U-Net [27] as the backbone architecture for $\Phi_{A}$, which comprises an encoder with four downsampling layers and a decoder with four upsampling layers. This subnetwork processes $I_{C0}$ to generate an anatomical knowledge representation $K_{A}$:
  (1) $$K_{A}=\Phi_{A}\left(I_{C0}\right).$$
- Lesion knowledge estimator ($\Phi_{L}$): We adopt the same architecture for $\Phi_{L}$ as used in $\Phi_{A}$. This subnetwork processes $I_{T2w}$ to extract lesion-related features, generating $K_{L}$:
  (2) $$K_{L}=\Phi_{L}\left(I_{T2w}\right).$$

These extracted representations are subsequently fused to form a comprehensive prior, encapsulating both anatomical context and lesion-specific features.

### 2.2. Knowledge Smoothing and Encoding

To ensure the spatial smoothness of the guidance signal and prevent high-frequency artifacts from being propagated to the generator, we introduce an explicit smoothing mechanism prior to feature encoding.

Gaussian smoothing layer: The prior semantic knowledge $K_{A}$ and $K_{L}$ is passed through a fixed nontrainable Gaussian smoothing layer. This operation, denoted as $G_{\sigma }$, convolves the feature maps with a Gaussian kernel characterized by a standard deviation σ. The smoothed priors $K_{A}$ and $K_{L}$ are obtained as follows:

(3) $${\tilde{K}}_{A}=G_{\sigma }\left(K_{A}\right),\;{\tilde{K}}_{L}=G_{\sigma }\left(K_{L}\right),$$

Here, the standard deviation for the Gaussian smoothing layer is set to σ=1.0. This operation effectively regularizes the content information, promoting smooth transitions that are essential for high-fidelity medical image synthesis.

Encoder (E): The smoothed priors ${\tilde{K}}_{A}$, ${\tilde{K}}_{L}$ and sequence $I_{C0}$ are concatenated and fed into the encoder, which consists of a series of strided convolutional layers. The encoder extracts a hierarchical content feature map $F_{C}$:

(4) $$F_{C}=E\left(concat\left[{\tilde{K}}_{A},I_{C0},{\tilde{K}}_{L}\right]\right),$$

where concat[·,·,·] denotes the concatenation operator. This feature map $F_{C}$ provides the foundational spatial information for the subsequent generation process.

### 2.3. Alias-Free Generator and Style Mapping Network

Alias free generator (G): Our generator G leverages the architectural design of StyleGAN3 [28]. The synthesis process is explicitly conditioned on the content features $F_{C}$ derived from our knowledge estimator. The generator is architected as a cascade of synthesis blocks, where each block sequentially performs alias-free upsampling, style modulation, and convolution and applies a filtered nonlinearity. Style modulation is achieved through adaptive instance normalization (AdaIN), where the style code w adjusts the statistics of the feature maps at each scale. This iterative refinement process culminates in the final high-fidelity image:

(5) $${\hat{I}}_{\mathrm{LGE}}=G\left(F_{C},w\right).$$

Style mapping network (M): The global visual characteristics of the synthesized image are governed by a style code w, which is produced by a dedicated style mapping network M. This network is implemented as a deep multilayer perceptron (MLP) with leaky ReLU activations and equalized learning rate scaling. It maps a latent vector z, sampled from a standard normal distribution N(0,I), to the more disentangled intermediate latent space:

(6) w=M(z).

The benefit of w lies in its ability to separate high-level visual attributes from one another. This provides independent control over stylistic factors crucial for clinical realism.

### 2.4. Loss Functions

KGSynth is trained using a composite objective function that combines a knowledge estimator constraint with adversarial, reconstruction, and perceptual losses to ensure high-fidelity and semantically accurate synthesis.

Knowledge estimator constraint loss: To ensure the extracted features are semantically meaningful, we introduce a segmentation constraint based on the Dice similarity coefficient. This loss is applied to force the two estimators to predict anatomical structure and lesion region priors:

(7) $$L_{\mathrm{constr}}=L_{\mathrm{Dice}}^{\mathrm{anatomy}}+L_{\mathrm{Dice}}^{\mathrm{lesion}},$$

Here each Dice loss term is defined as

(8) $$L_{\mathrm{Dice}}=1-\frac{2|\hat{M}\cap M|+\epsilon }{|\hat{M}|+|M|+\epsilon },$$

where $\hat{M}$ and M denote the predicted and ground truth segmentation masks, respectively, and ϵ is a small constant for numerical stability. The knowledge estimators ($\Phi_{A}$ and $\Phi_{B}$) are designed to function as automatic segmentation modules. For the cardiac task, the ground truth mask (M) is obtained from the challenge-provided manual annotations, where anatomical structure labels (myocardium and left ventricular cavity) are used to supervise the anatomical estimator ($\Phi_{A}$) and lesion labels (edema) are used to supervise the lesion estimator ($\Phi_{B}$). For the brain tumor task, ground truth tumor masks are manually delineated by two board-certified neuroradiologists. In this setting, the anatomical estimator ($\Phi_{A}$) is omitted, and tumor labels are used to supervise the lesion estimator ($\Phi_{B}$).

Adversarial and regularization loss: To enforce realism in the synthesized images, we employ a non-saturating adversarial loss coupled with R1 gradient penalty regularization [29]. The adversarial loss for the generator (G) aims to maximize the discriminator’s (D) belief in the generated images:

(9) $$L_{\mathrm{adv},\;G}=-E_{z}\left[\log\left(D\left(G\left(F_{C},w\right)\right)\right)\right].$$

Reconstruction and perceptual losses: To maintain content fidelity and perceptual realism, we employ a combination of L1 reconstruction and VGG perceptual losses [30].

The L1 reconstruction loss measures the pixel-wise absolute difference between the synthesized image ${\hat{I}}_{\mathrm{LGE}}$ and the ground truth $I_{\mathrm{LGE}}$, ensuring anatomical consistency:

(10) $$L_{\mathrm{rec}}={∥{\hat{I}}_{\mathrm{LGE}}-I_{\mathrm{LGE}}∥}_{1}.$$

The perceptual loss encourages perceptual similarity by comparing high-level features extracted by a pretrained VGG-19 network. It is calculated as the L1 distance between the feature maps of the synthesized and ground truth images:

(11) $$L_{\mathrm{perc}}=\sum_{i}{∥\phi_{i}\left({\hat{I}}_{\mathrm{LGE}}\right)-\phi_{i}\left(I_{\mathrm{LGE}}\right)∥}_{1},$$

where $\phi_{i}$ denotes the feature map from the *i*-th layer of the VGG-19 network.

Loss function for generator: The objective function for optimizing the generator and the knowledge estimator is a weighted sum of the aforementioned components:

(12) $$L_{G}=\lambda_{\mathrm{adv}}L_{\mathrm{adv},\;G}+\lambda_{\mathrm{rec}}L_{\mathrm{rec}}+\lambda_{\mathrm{perc}}L_{\mathrm{perc}}+\lambda_{\mathrm{constr}}L_{\mathrm{constr}},$$

where the $\lambda_{*}$ terms are hyperparameters that balance the contribution of each loss.

Loss function for discriminator: The discriminator is trained separately using its own objective function, which includes the adversarial term

(13) $$L_{\mathrm{adv},\;D}=-E_{x∼p_{I_{LGE}}}D\left(x\right)-E_{x∼p_{{\hat{I}}_{LGE}}}\left(1-D\left(x\right)\right).$$

To stabilize discriminator training and prevent its gradients from exploding, we apply the $L_{R1}$ term, which penalizes the gradient norm of the discriminator’s output with respect to real images:

(14) $$L_{R1}=\frac{\gamma }{2}E_{I_{\mathrm{LGE}}∼p_{\mathrm{data}}\left(I_{\mathrm{LGE}}\right)}[∥\nabla_{I_{\mathrm{LGE}}}D\left(I_{\mathrm{LGE}}\right){∥}^{2}],$$

where γ is a regularization hyperparameter. Consequently, the final objective for the discriminator is defined as follows:

(15) $$L_{D}=L_{\mathrm{adv},\;D}+L_{R1}.$$

Optimization strategy: Instead of minimizing a single static loss, KGSynth adopts an alternating optimization strategy. The generator and discriminator are updated iteratively to solve the minimax game:

(16) $$\min_{G,\Phi }\max_{D}\left(L_{G},L_{D}\right),$$

where Φ denotes the parameters of the knowledge estimator. In each training step, we first update the discriminator to minimize $L_{D}$ and subsequently update the generator and knowledge estimator to minimize $L_{G}$.

## 3. Experiments

To validate the performance, robustness, and versatility of KGSynth, a comprehensive evaluation was conducted on two distinct and clinically challenging synthesis tasks. One task involves the synthesis of cardiac LGE images, and the other concerns the synthesis of brain CBV maps.

### 3.1. Datasets and Preprocessing

#### 3.1.1. Cardiac LGE Synthesis Dataset

Cardiac data from the CARE2024 MyoPS Challenge was used to evaluate the proposed method. This dataset comprises multi-sequence MRI images from 145 patients with myocardial infarction, each patient having co-registered C0, T2w, and LGE sequence images. Among these multi-sequence images, the C0 sequence primarily provides anatomical and functional information, while the T2w sequence offers additional pathological insights, specifically, hyperintense regions caused by edema that typically correspond spatially to acute or subacute myocardial scar tissue. Manual ground truth masks provided by the CARE2024 MyoPS Challenge include annotations of the left ventricle (LV) cavity, right ventricle (RV) cavity, LV myocardium (MYO), scar, and edema; in this study, only the LV, MYO, and edema annotations were utilized. The core objective is to synthesize high-fidelity LGE images from these non-contrast-enhanced input images, thereby enabling precise delineation of scar tissue. Given that scar signatures are highly inconspicuous prior to contrast enhancement, this task is undoubtedly very challenging.

#### 3.1.2. Brain CBV Map Synthesis Dataset

To further evaluate the versatility and flexibility of the proposed framework, a second private dataset was utilized. This dataset comprises retrospectively collected imaging data from 60 patients with pathologically confirmed brain gliomas. For each patient, three imaging modalities were available: pre-contrast T1-weighted images providing anatomical reference, pre-contrast ADC maps characterizing tumor cellularity via diffusion restriction, and contrast-enhanced CBV maps, which serve as the gold standard for tumor perfusion assessment. Detailed acquisition parameters for the MRI sequences are provided in the Supplementary Materials. The demographic and clinical characteristics of this cohort are summarized in Table 1.

Only cases with complete imaging sequences were included, while scans affected by severe artifacts or missing modalities were excluded. The study protocol was approved by the institutional ethics committee, and all data were anonymized prior to analysis in accordance with data protection regulations.

The ground truth masks for brain tumors were delineated by two board-certified neuroradiologists, each with more than five years of experience in neuroimaging diagnosis. All cases were independently annotated by both readers to ensure consistency and reproducibility. In cases of disagreement, the readers jointly reviewed the images and reached a consensus through discussion. Inter-observer agreement analysis demonstrated good consistency between the two annotators.

As CBV maps are essential for tumor grading, biopsy guidance, and treatment planning, this dataset was used to evaluate the model’s capability to synthesize quantitatively accurate CBV maps from non-contrast-enhanced MRI sequences.

#### 3.1.3. Data Preprocessing

During data preprocessing, both datasets underwent similar operations. All image slices were resampled to a uniform inplane resolution of 1.25×1.25 ${\mathrm{mm}}^{2}$, and image intensities were normalized to the range [0, 1]. For the cardiac dataset, images were first cropped around the region of interest (ROI) centered on the heart and then resized to 224 × 224 pixels. In contrast, for the brain dataset, images were directly resized to 224 × 224 pixels. Detailed MRI acquisition parameters are cataloged in Table 2.

### 3.2. Evaluation and Implementation Details

#### 3.2.1. Quantitative Metrics

We employed two standard metrics for image fidelity assessment: peak signal-to-noise ratio (PSNR) for pixel-level accuracy and the structural similarity index measure (SSIM) to evaluate perceptual and structural correctness. To ensure statistical rigor and avoid inflation of significance due to slice-level correlations, we performed a patient-level evaluation. While metrics were initially calculated for each 2D slice, they were subsequently averaged across all slices belonging to the same patient to derive a single representative value per subject. Consequently, the unit of analysis is the patient (N = 29 for the cardiac test set; N = 12 for the brain test set). Statistical comparisons between KGSynth and baseline methods were performed on these patient-level aggregates using the Wilcoxon signed-rank test.

#### 3.2.2. Clinical Evaluation

For the brain CBV map synthesis task, a blinded reader study was conducted. Two board-certified neuroradiologists (i.e., with 8 and 12 years of experience) independently evaluated the synthetic maps from the test set in a randomized order, without knowledge of whether the images were real or synthesized. They assessed (1) overall image quality on a 5-point Likert scale (1 = poor and 5 = excellent) and (2) diagnostic consistency, where they scored the synthetic map’s ability to correctly identify the location and extent of high-perfusion tumor regions compared to the ground truth (0 = misleading, 1 = inconclusive, and 2 = perfectly consistent). Inter-rater reliability for both metrics was quantified using the Intraclass Correlation Coefficient (ICC).

#### 3.2.3. Implementation and Baselines

The model was implemented in PyTorch 1.13.0 and trained for 3000 epochs on a single NVIDIA RTX 4090 GPU with a batch size of 8. We performed a patient-level data split (i.e., 80% train and 20% test) to prevent data leakage. KGSynth was implemented following the principles outlined in [28]. We used the Adam optimizer with a learning rate of $1\times 10^{-4}$ for the generator and $4\times 10^{-4}$ for the discriminator, with $\beta_{1}=0.0$ and $\beta_{2}=0.99$. The R1 regularization weight γ was set to 10. Loss weight hyperparameters $\lambda_{\mathrm{adv}}$, $\lambda_{\mathrm{rec}}$, $\lambda_{\mathrm{perc}}$, $\lambda_{\mathrm{constr}}$ were set to 1, 0.5, 0.5, 1. During the inference phase, the latent vector z was randomly sampled from a standard normal distribution N(0,I)

### 3.3. Results

#### 3.3.1. Performance of Cardiac LGE Synthesis

To validate the efficacy of the proposed method, we compared KGSynth against four representative baselines: VNE-Net [21] and PSCGAN [26], which are specialized for cardiac synthesis, and two general-purpose image-to-image translation models, CycleGAN [18] and Pix2pix [17].

- VNE-Net [21]: A domain-specific network designed for the exact task of cardiac LGE synthesis.
- PSCGAN [26]: A lesion-aware model that uses a progressive strategy for myocardial scar synthesis.
- CycleGAN [18]: A foundational framework for unpaired image-to-image translation, included to benchmark performance without paired supervision.
- Pix2pix [17]: A seminal conditional GAN for paired image translation. We used a channel-wise concatenation of non-contrast inputs to provide it with equivalent information to our model.

To ensure a fair comparison, all models were trained and evaluated on the same datasets using identical patient-level train/test splits and input preprocessing. For methods that support multi-channel inputs (e.g., Pix2pix), the same non-contrast MRI sequences were provided via channel-wise concatenation, while domain-specific models (e.g., VNE-Net and PSCGAN) were trained using their official implementations and recommended training settings to reflect their optimal performance.

As shown in Table 3, KGSynth significantly outperforms all baseline methods in both SSIM and PSNR metrics for cardiac LGE synthesis, demonstrating substantial improvements over existing task-specific and general-purpose models. Most notably, in terms of structural fidelity, KGSynth achieved an SSIM of 0.567 (95% CIs [0.542, 0.592]). This represents a substantial margin of improvement approximately 9.9% (p<0.01, r = 0.452 (effect size *r* derived from the Wilcoxon signed-rank test, calculated as $r=Z/\sqrt{N}$)) higher than the second best-performing method, Pix2pix (0.516), and significantly surpassing the domain-specific VNE-Net (0.473, p<0.001, r = 0.576). This significant performance gap demonstrates that KGSynth effectively leverages lesion prior information extracted from T2w images to synthesize high-fidelity pathological structures. In terms of pixel-level accuracy, our method achieves a PSNR of 19.48 dB (95% CIs [18.84, 20.12]), surpassing the second-best approach (VNE-Net) by more than 2.0 dB (p<0.001, r = 0.596). Moreover, KGSynth exhibits lower standard deviations (SSIM ± 0.066; PSNR ± 1.69), indicating superior robustness and stability in consistently generating high-quality images. In contrast, unsupervised methods such as CycleGAN perform the worst (14.15 dB), highlighting the critical importance of explicit knowledge guidance in synthesis tasks.

**Figure 2:**
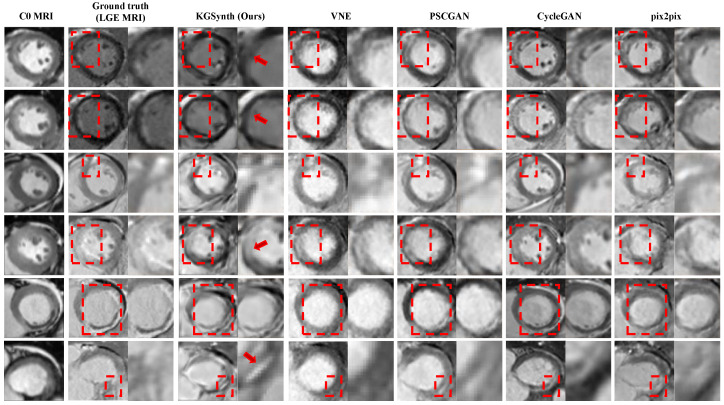
Visual comparison of cardiac LGE synthesis methods. The red rectangle in the figure highlights the advantages of KGSynth over other methods in scar areas.

The visual results in Figure 2 provide an intuitive illustration of the aforementioned data superiority. Existing methods such as VNE-Net and PSCGAN produce images that are very blurry and fail to fully depict the complete extent of myocardial scars. In contrast, the LGE images generated by KGSynth are remarkably clear, with scar tissue contours that are well-defined and highly consistent with real images. Additionally, it is worth noting that methods like CycleGAN and Pix2pix either completely miss the lesions or generate significant image artifacts. These comparisons demonstrate that KGSynth can leverage lesion priors from T2w images to accurately generate critical pathological features.

#### 3.3.2. Ablation Study for Knowledge

To verify that lesion prior knowledge is crucial for accurately synthesizing pathological images, we conducted an ablation study. We designed a variant of KGSynth that uses only the C0 sequence as input, excluding the lesion prior information provided by the T2w image. This variant is denoted as KGSynth (w/o prior).

The results in Table 4 highlight the advantages of our approach. The full model, which incorporates explicit prior knowledge, achieves superior performance. Its SSIM improves from 0.508 to 0.567 and PSNR increases from 18.37 dB to 19.48 dB. Crucially, the full model demonstrates greater stability, with a reduced SSIM standard deviation (±0.066 vs. ±0.098). This indicates that the knowledge prior not only boosts image quality but also constrains the model’s generative space, preventing it from producing structurally implausible artifacts.

**Figure 3:**
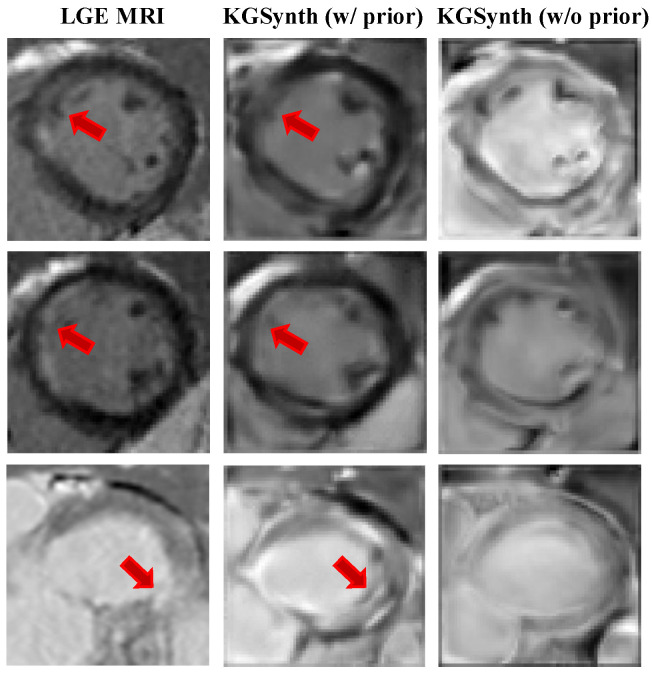
Ablation study on knowledge priors. The **left** column displays the ground truth LGE MRI. The **middle** column shows the result of KGSynth with explicit knowledge priors, accurately capturing the hyperintense scar tissue. The **right** column shows the result without priors, where the model fails to synthesize the lesion, resulting in a generic appearance. The red arrow indicates the region generated with high fidelity under the guidance of the prior.

The visual comparison in Figure 3 further supports these conclusions. As shown in the KGSynth (w/o prior) column, the lack of lesion prior information from the T2w image prevents it from synthesizing accurate pathological features. Furthermore, this model generates ordinary-looking myocardial images, with key scar tissue either missing or very blurry. In contrast, the full model KGSynth (w/ prior) accurately generates high-signal myocardial scars. It fully preserves both the complete extent of the scar and the normal structure of the surrounding myocardium. This confirms that prior knowledge derived from multi-sequence inputs can generate pathological features with high fidelity.

#### 3.3.3. Ablation Study for Gaussian Smoothing Layer

A Gaussian smoothing layer was designed to regularize the prior information and prevent high-frequency input noise from propagating into the generator. To investigate the effectiveness of this Gaussian smoothing layer, we conducted an ablation study by removing it.

As shown in Table 5, removing the Gaussian smoothing layer resulted in a performance drop. The SSIM decreased from 0.567 to 0.531, and the PSNR fell from 19.48 dB to 19.02 dB. Furthermore, the model without smoothing layer had a higher SSIM standard deviation (±0.083 vs. ±0.066), indicating that smoothing contributes to increased stability and robustness during the synthesis process. This ablation study confirms that the smoothing layer ensures the generator receives clear, spatially coherent signals, thereby producing high-fidelity images without artifacts.

**Figure 4:**
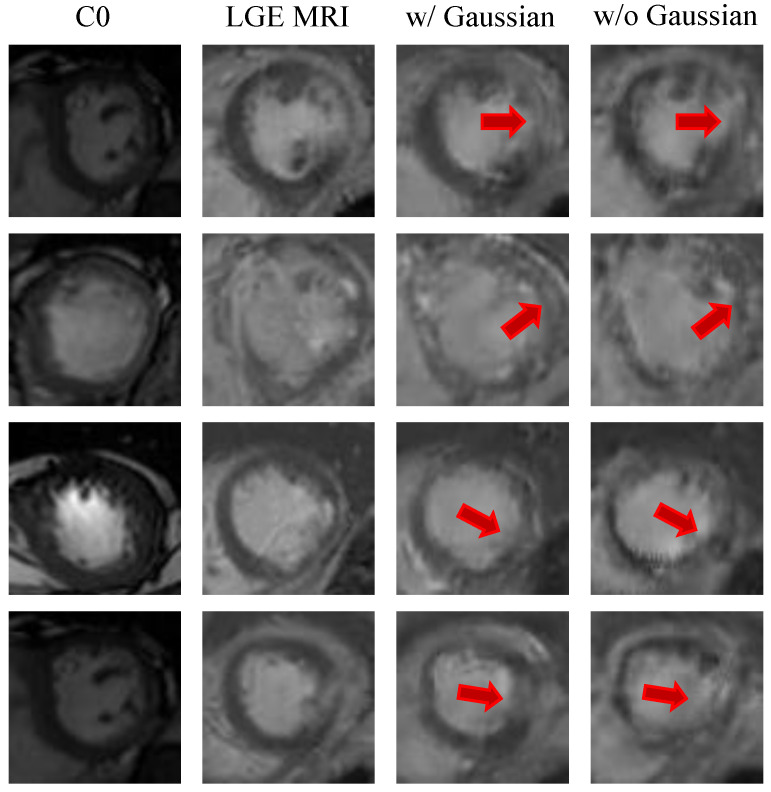
Qualitative comparison of C0, LGE (ground truth), and synthesized LGE images with and without the Gaussian smoothing layer. Red arrows indicate regions where the Gaussian smoothing layer ensures sharper myocardial boundaries and better-preserved scar tissue, effectively suppressing high-frequency artifacts to improve clinical readability compared to the variant without smoothing.

As shown in the visual comparison in Figure 4, removing Gaussian smoothing severely degrades the structural quality of the synthesized images. The model without the Gaussian smoothing layer produces blurred myocardial boundaries and discontinuous cardiac ring structures and loses some fine scar textures, as indicated by the red arrows in the figure. In contrast, the model with Gaussian smoothing accurately recovers both endocardial and epicardial contours and retains subtle intensity variations that are close to real LGE images. These results consistently demonstrate the importance of processing the prior with a Gaussian smoothing layer. It stabilizes the synthesis process and ensures that the generated image is anatomically and pathologically plausible.

#### 3.3.4. Performance of Brain CBV Map Synthesis

To validate the versatility and flexibility of our framework, we extended KGSynth to a new brain tumor task, synthesizing quantitative CBV maps from non-contrast T1w and ADC MRI sequences. Note that although CBV maps were normalized to the range [0, 1] prior to model training, the synthesized results preserve quantitative information regarding relative perfusion values and spatial distribution patterns within clinically relevant regions. Unlike the cardiac task, this application requires the model to capture complex perfusion dynamics and generate heterogeneous tumor textures.

Despite using non-contrast images as input, our method achieved robust quantitative results on brain datasets. KGSynth achieved a mean SSIM of 0.697±0.057 (95% CIs [0.661, 0.733]) and a mean PSNR of 24.49±1.40 dB (95% CIs [23.60, 25.38]). These metrics demonstrate a high degree of structural similarity and pixel-level fidelity between virtual and real CBV maps. To further evaluate the quantitative accuracy in clinically relevant areas, we calculated the Mean Absolute Error (MAE) in the tumor regions of interest (ROIs). Our method achieved a low ROI MAE of 0.0801 ± 0.0366, indicating that the synthesized perfusion values are highly consistent with the ground truth in lesion areas. This indicates that our knowledge-guided model effectively learns the latent correspondence between unenhanced images and enhanced maps.

**Figure 5:**
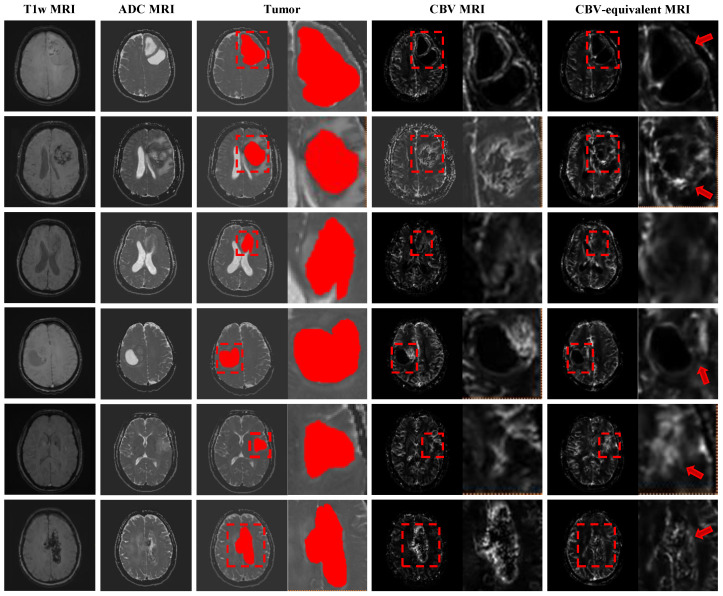
Expansion to brain CBV map synthesis. KGSynth generates a virtual CBV map that accurately delineates the high-perfusion tumor boundaries (red boxes), preserving internal tumor heterogeneity and texture. This shows strong visual and quantitative correspondence with the ground truth, facilitating the identification of clinical perfusion hotspots. The red arrow indicates that the tumor boundary generated by our method is sharper and the tumor region is more faithful.

Figure 5 clearly demonstrates the diagnostic potential of this model. Our synthesized CBV map closely matches the real CBV map, especially in the tumor region.

(1) Tumor Fidelity: The model successfully reconstructs the unique, non-uniform intensity signal characteristic of brain tumors. It accurately identifies and generates high-perfusion areas, which helps in determining tumor grade and planned biopsy location.
(2) Detail Preservation: As shown by the red arrow in the magnified area of the figure, KGSynth preserves fine structural details and clear boundaries, avoiding the image over-smoothing and artifacts common in such tasks.

This successful application demonstrates the effectiveness of KGSynth’s core mechanism. By utilizing explicit anatomical and lesion prior information, this capability is not only applicable to cardiac MRI but also applicable to other organs and functional imaging modalities.

#### 3.3.5. Clinical Evaluation of Synthesized CBV

**Figure 6:**
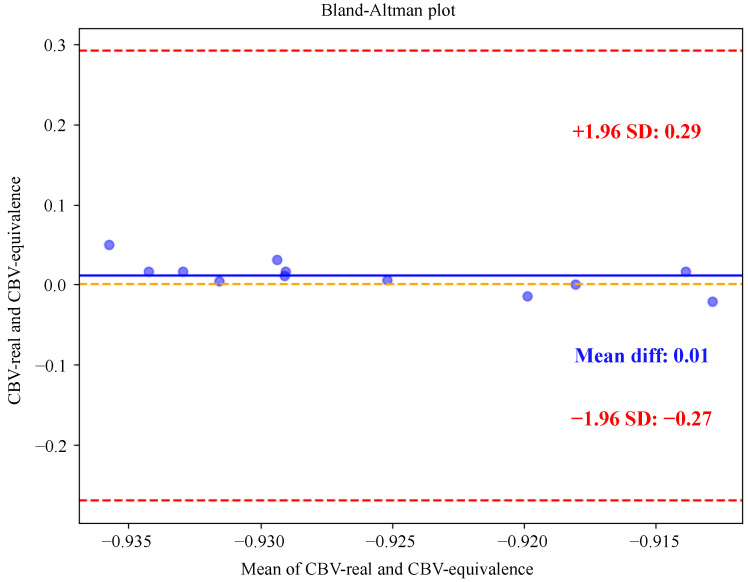
Bland–Altman plot comparing quantitative CBV values extracted from real and synthetic maps. The plot shows strong agreement with a mean difference close to zero and narrow 95% limits of agreement, indicating no systematic bias. This high quantitative consistency suggests that the synthesized maps have potential for clinical perfusion assessment.

To evaluate the clinical diagnostic value of the synthesized images, we conducted a series of image quality evaluations. Neuroradiologists rated the synthesized CBV maps as high-quality, with a good agreement in scores among multiple physicians (ICC = 0.82). When used for diagnosis, the synthesized images achieved a high ICC score of 0.835, indicating that the synthesized CBV maps reliably identified the size and location of hyperperfused tumor regions. The Bland–Altman plot in Figure 6 further corroborates this finding, showing a high quantitative agreement between real and synthesized CBV values, with minimal bias and narrow limits of agreement. These results confirm that KGSynth is capable of producing not only visually correct images but also quantitatively accurate images suitable for diagnostic purposes.

## 4. Discussion

The experimental results show that the proposed KGSynth method performs well in image synthesis tasks. For cardiac LGE synthesis, our method achieved superior performance, with an SSIM of 0.567 and a PSNR of 19.48 dB. For brain CBV map synthesis, it achieved an SSIM of 0.697 and a PSNR of 24.49 dB, demonstrating the method’s good versatility. These results suggest that KGSynth could serve as a useful tool for assessing myocardial viability and tumor perfusion, without the risks associated with contrast agents.

Component analysis validated the contribution of each module within KGSynth. Optimal performance was achieved by coupling the knowledge estimator with Gaussian smoothing. Mechanistically, the knowledge estimator disentangles critical anatomical and pathological features from non-contrast inputs, effectively mitigating the generation of artifactual lesions. Concurrently, the Gaussian smoothing layer suppresses high-frequency input noise to maintain spatial coherence. The synergy between these components enables the synthesis of high-fidelity images with diagnostic reliability.

The automated generation of virtual contrast images can be integrated into clinical protocols to address a critical unmet need for patients with renal impairment or those requiring longitudinal monitoring. By enabling the quantification of myocardial scarring and perfusion without the safety constraints of GBCAs, KGSynth offers a safer diagnostic pathway that complements standard non-contrast acquisition workflows.

Contrast-enhanced MRI is affected by injection timing, dose, and patient-specific physiology, resulting in variability in enhancement patterns. This variability is implicitly represented in the training data, allowing the model to learn a statistical mapping consistent with routine clinical acquisitions. In the proposed framework, contrast-related appearance is learned through style modulation, while anatomical and lesion-related information is derived from non-contrast sequences. Consequently, evaluation metrics and clinical assessments reflect the model’s ability to reproduce contrast-dependent patterns under clinically realistic heterogeneity.

Despite these findings, several limitations merit consideration. (1) In clinical practice, variations in magnetic field strength, scanner vendors, and acquisition protocols can introduce domain shifts that may affect synthesis performance. Therefore, although effective on our test sets, the model lacks validation on large-scale, multi-center datasets. Future work will assess robustness generalizability against inter-site variability arising from different scanner vendors and populations. (2) The brain CBV synthesis experiments were conducted on a private dataset, which limits reproducibility. In addition, clinical evaluation involved a limited number of experts (two neuroradiologists). Although high inter-rater reliability was achieved, future studies will validate KGSynth on larger, multi-center, and publicly available brain MRI datasets and include a broader panel of readers with varying experience to improve reproducibility, external validity, and clinical generalizability. (3) While this study utilized Bland–Altman plots and expert clinical scoring to assess brain lesion fidelity, the primary quantitative evaluations relied on image-level metrics (SSIM and PSNR). Future work will aim to validate the clinical utility of the synthesized images by using them as inputs for automated segmentation algorithms. This will allow for the calculation of lesion-specific metrics, such as the Dice similarity coefficient and lesion-wise error, to provide a more rigorous assessment of pathological accuracy. (4) Due to limited spatial resolution and reduced contrast in non-contrast inputs, subtle lesion enhancement or small-vessel pathology may be underestimated. In addition, highly heterogeneous tumor perfusion poses challenges for accurate synthesis. These factors may lead to localized discrepancies between synthesized images and real contrast-enhanced images. Future work will require higher-resolution inputs, more physiology-aware modeling, and validation on more diverse clinical cohorts.

## 5. Conclusions

In this paper, we introduced a virtual image synthesis framework named KGSynth. It uses knowledge guidance to synthesize diagnostic images, such as cardiac LGE images and brain CBV maps, from non-contrast MRI. KGSynth incorporates a lesion prior estimator and a Gaussian smoothing layer to address the difficulty in accurately generating lesion areas. Extensive evaluations on cardiac and brain data demonstrate that KGSynth not only achieves high-fidelity synthesis quality but also produces results of significant clinical diagnostic value. This work provides an effective tool for obtaining critical diagnostic information in clinical scenarios where the use of GBCAs is constrained. Future work will focus on enhancing robustness to inter-site variability, validating the framework on larger multi-center and public datasets, and expanding clinical reader studies. We will also explore task-based validation, higher-resolution inputs, and physiology-aware modeling to better capture subtle lesions and heterogeneous perfusion for clinical integration.

## Figures and Tables

**Table 1:** Demographic and clinical characteristics of the study cohort.

Parameter	Study Cohort (Total)
No. of patients	60
Mean age (years) ± SD	38.2 ± 19.8
Sex, n (%)	
Male	35 (58.3%)
Female	25 (41.7%)
Population Type, n (%)	
Adult	46 (76.7%)
Pediatric	14 (23.3%)
Pathological Grade (WHO)	
WHO 1	6 (10.0%)
WHO 2	16 (26.7%)
WHO 3	13 (21.7%)
WHO 4	20 (33.3%)
Undefined *	5 (8.3%)

Note that values are presented as mean ± standard deviation or count (percentage). WHO: World Health Organization. Undefined *: Cases with incomplete pathological grading.

**Table 2:** MRI acquisition parameters.

Sequence/Map	SPC (mm)	Slice Num	Resolution (${\mathrm{mm}}^{2}$)
**Cardiac MRI**			
C0	10∼23	2∼9	1.19 × 1.19∼2.13 × 2.13
T2w	10∼23	2∼9	0.73 × 0.73∼2.24 × 2.24
LGE	10∼23	2∼9	0.73 × 0.73∼1.86 × 1.86
**Brain MRI**			
T1w	5∼7	20∼24	0.34 × 0.34∼0.57 × 0.57
ADC	5∼7	20∼24	0.34 × 0.34∼0.57 × 0.57
CBV	5∼7	20∼24	0.34 × 0.34∼0.57 × 0.57

**Table 3:** Quantitative comparison on the cardiac LGE synthesis task. Values are presented as mean ± std. The best results are highlighted in bold. Statistical significance assessed via the Wilcoxon signed-rank test (N = 29) with Bonferroni correction. Here, * is for p<0.01 and ** for p<0.001.

Method	SSIM ↑	PSNR (dB) ↑
VNE-Net [21]	0.473 ± 0.105 **	17.40 ± 1.94 **
PSCGAN [26]	0.468 ± 0.091 **	17.31 ± 2.06 **
CycleGAN [18]	0.496 ± 0.083 **	14.15 ± 2.21 **
Pix2pix [17]	0.516 ± 0.079 *	15.73 ± 2.13 **
**KGSynth (Ours)**	**0.567 ± 0.066**	**19.48 ± 1.69**

**Table 4:** Quantitative ablation study on the impact of knowledge priors for cardiac LGE synthesis. Values are presented as mean ± std. The best results are highlighted in bold.

Knowledge	SSIM ↑	PSNR (dB) ↑
KGSynth (w/o prior)	0.508 ± 0.098	18.37 ± 2.23
KGSynth (w/ prior)	**0.567 ± 0.066**	**19.48 ± 1.69**

**Table 5:** Ablation of the Gaussian smooth layer in KGSynth. Values are presented as mean ± std. The best results are highlighted in bold.

Method	SSIM ↑	PSNR (dB) ↑
KGSynth (w/o Gaussian Smooth Layer)	0.531 ± 0.083	19.02 ± 1.81
KGSynth (w/ Gaussian Smooth Layer)	**0.567 ± 0.066**	**19.48 ± 1.69**

## Data Availability

Cardiac MRI dataset: CARE2024-MyoPS Challenge, https://zmic.org.cn/care_2024/track4/ (accessed on 24 December 2024). Brain MRI dataset: The datasets are not publicly available due to restrictions of the hospital.

## Supplementary Materials

The following supporting information can be downloaded at https://www.mdpi.com/article/10.3390/diagnostics16040576/s1, Table S1: T1-Weighted Imaging; Table S2: T2-Weighted Imaging; Table S3: Precontract and Postcontrast T1-Weighted Imaging; Table S4: T2-Weighted Fluid-Attenuated Inversion Recovery Imaging; Table S5: Susceptibility Weighted Imaging-Maximum Intensity Projection; Table S6: Diffusion-Weighted Imaging-Apparent Diffusion Coefficient Sequence.
