# Supplementary material for: Knowledge-Guided Framework for Synthesizing Contrast-Dependent Data from Multi-Sequence Non-Contrast MRI
Source: Diagnostics (Basel). 2026 Feb 14;16(4):576. doi: 10.3390/diagnostics16040576 (PMC12940084; doi:10.3390/diagnostics16040576)
Supplement: Supplementary file 1 [file diagnostics-16-00576-s001.zip › diagnostics-4109245-supplementary.pdf]

MRI Parameters

All patients were imaged on a 3T or 1.5T MRI scanner (Siemens Healthiness) with the following detailed parameters:

Table S1：T1-Weighted Imaging (T1WI)

Parameter	Internal data	External data
repetition time (TR)	250 ms	400 ms ~ 2200 ms
echo time (TE)	2.46 ms	2.46 ms ~ 17 ms
field of view (FOV)	220mm	240mm
slice thickness	5.0 mm	0.8 mm ~ 5.0 mm
distance factor	120%	N/A
flip angle (FA)	70°	8° ~ 90°
spacing	0.6875mm × 0.6875mm × 5.0 mm	1.000mm × 1.000mm × 1.000mm

Note. N/A: MRI scan parameters information not provided by the external dataset.

Table S2：T2-Weighted Imaging (T2WI)

Parameter	Internal data	External data
repetition time (TR)	6000 ms	3200 ms ~ 6880 ms
echo time (TE)	125 mm	84 mm ~ 485 mm
field of view (FOV)	220 mm	240 mm
slice thickness	5 mm	0.9 mm ~ 5 mm
distance factor	120%	N/A
flip angle (FA)	90°	90° ~ 180°
voxel size	0.5729mm× 0.5729mm × 5.0 mm	1.000mm × 1.000mm × 1.000mm

Note. N/A: MRI scan parameters information not provided by the external dataset.

Table S3：Precontract and Postcontrast T1-Weighted Imaging (T1_C)

Parameter	Internal data	External data
repetition time (TR)	250 mm	1180 mm ~ 2200 mm
echo time (TE)	2.46 mm	2.46mm ~ 3.87 mm
field of view (FOV)	220 mm	240mm
slice thickness	5.00 mm	0.90 mm ~ 1.00 mm
distance factor	120%	N/A
flip angle (FA)	70°	8° ~ 15°
voxel size	0.6875mm × 0.6875mm × 5.0mm	1.000mm × 1.000mm × 1.000mm

Note. N/A: MRI scan parameters information not provided by the external dataset.

Table S4：T2-Weighted Fluid-Attenuated Inversion Recovery Imaging (T2_FLAIR)

Parameter	Internal data	External data
repetition time (TR)	8500 mm	8000 mm~ 15830 mm
echo time (TE)	81 mm	75 mm~ 141 mm
field of view (FOV)	199.4mm ~ 220.00 mm	240 mm

slice thickness	5 mm	3 mm ~ 4 mm
distance factor	120%	N/A
flip angle (FA)	150°	120° ~ 170°
voxel size	0.6875mm × 0.6875mm × 5.0 mm	1.000mm × 1.000mm × 1.000mm

Note. N/A: MRI scan parameters information not provided by the external dataset.

Table S5：Susceptibility Weighted Imaging-Maximum Intensity Projection (SWI-MIP)

Parameter	Internal data	External data
repetition time (TR)	27 mm	N/A
echo time (TE)	20 mm	N/A
field of view (FOV)	172.5 mm ~ 240mm	N/A
slice thickness	16	N/A
distance factor	N/A	N/A
flip angle (FA)	15°	N/A
voxel size	0.8984mm× 0.8984mm × 16 mm~ 0.9375mm× 0.9375mm × 16 mm	N/A

Note. N/A: MRI scan parameters information not provided by the external dataset.

Table S6：Diffusion-Weighted Imaging-Apparent Diffusion Coefficient Sequence (DWI-ADC)

Parameter	Internal data	External data
repetition time (TR)	4360 mm ~ 4600 mm	3100 mm ~ 8000 mm
echo time (TE)	58 mm ~65 mm	83 mm ~ 121 mm
field of view (FOV)	200mm	240mm
slice thickness	5 mm	2.5 mm ~ 5 mm
distance factor	120%	N/A
flip angle (FA)	180°	90°
voxel size	0.982mm× 0.982mm × 5.2 mm ~ 1.250mm× 1.250mm × 6.5 mm	1.000mm × 1.000mm × 1.000mm

Note. N/A: MRI scan parameters information not provided by the external dataset.
